# Assessment of the Syndemic Relationship Between Individual, Social, and Structural Determinants of Tuberculosis Among People Living in Johannesburg, South Africa

**DOI:** 10.3390/ijerph22081272

**Published:** 2025-08-14

**Authors:** Fiona Tsungirai Tanyanyiwa, Renay Helouise Van Wyk, Keitshepile Geoffrey Setswe

**Affiliations:** 1 Department of Environmental Health, Faculty of Health Sciences, University of Johannesburg, Johannesburg 2006, South Africa; tfionatsungi2@gmail.com; 2 Implementation Research Division, Aurum Institute, Johannesburg 2193, South Africa; gsetswe@auruminstitute.org; 3 Department of Health Studies, University of South Africa, Pretoria 0003, South Africa

**Keywords:** tuberculosis, syndemic theory, social determinants, behavioural risk factors, structural barriers

## Abstract

Tuberculosis (TB) remains a critical public health issue in Johannesburg, South Africa, driven by a complex interplay of individual, social, and structural factors. This study assessed the syndemic relationship between these determinants to understand their collective impact on TB burden and treatment outcomes. A cross-sectional survey was conducted among TB patients attending selected clinics, examining behavioural risks (e.g., smoking, alcohol use, HIV co-infection), social conditions (poverty, overcrowding, stigma), and structural challenges (access to healthcare, migration status). The results revealed a significant co-occurrence of TB and HIV (56.1%), alongside high rates of smoking (33.1%) and alcohol use (45.2%). Unemployment (50.2%), inadequate housing, and limited healthcare access, particularly for undocumented migrants (26.2%), were also prominent. Factor analysis demonstrated a syndemic interaction between behavioural and social determinants, underscoring the compounded vulnerability of affected populations. The findings highlight the necessity of integrating medical interventions with social and structural reforms. Recommendations include TB-HIV co-management, substance abuse programmes, improved housing, and inclusive healthcare access. A multisectoral approach addressing both health and socioeconomic inequalities is critical for comprehensive TB control in urban South African contexts.

## 1. Introduction

Tuberculosis (TB) remains one of the most pressing global public health challenges, despite being a preventable and curable disease. According to the World Health Organization [1], approximately 10.6 million people developed TB in 2022, and 1.3 million died from the disease. These deaths are overwhelmingly concentrated in low- and middle-income countries, where poverty, undernutrition, HIV, and systemic health inequities create conditions for ongoing transmission and poor outcomes. TB not only surpasses HIV and malaria in global mortality, but it also disproportionately affects individuals from disadvantaged and marginalised populations who are often exposed to a wide range of health-compromising conditions [2].

South Africa, in particular, bears one of the heaviest TB burdens globally. The 2022 TB incidence was estimated at 513 per 100,000 people, with over 280,000 new cases recorded that year [1]. Despite ongoing national control efforts, South Africa accounted for 3.3% of the global TB burden. In addition, 48% of TB patients in 2022 were co-infected with HIV, making TB the leading cause of death among people living with HIV in the country [3]. In 2023, the country was ranked among the 30 high-TB burden countries by the World Health Organization [4]. TB patterns in South Africa also exhibit urban–rural disparities. While rural provinces such as KwaZulu-Natal and the Eastern Cape tend to report the highest overall TB incidence, urban centres like Johannesburg experience a disproportionate burden of TB-HIV co-infection, compounded by high levels of migration, housing shortages, population density, and health service bottlenecks [4].

A growing body of evidence now recognises that TB is not only a biomedical condition but a socially and structurally driven inequity, which is shaped by the environments in which people are born, live, work, and age [5]. These structural determinants such as poverty, food insecurity, inadequate housing, unemployment, limited education, and migration can influence both the risk of TB infection and the effectiveness of prevention and treatment strategies. At the individual level, behavioural and biological risk factors, including HIV status, smoking, alcohol use, substance abuse, and malnutrition, further compound susceptibility to TB [6,7].

The concept of syndemics has gained increasing relevance in TB research. A syndemic refers to the synergistic interaction of two or more co-existing diseases or conditions in a population, which is exacerbated by social and structural inequities, leading to worse health outcomes [8]. In the South African context, the syndemic interaction between TB, HIV/AIDS, and other non-communicable or social conditions, such as substance abuse, undernutrition, food insecurity, stigma, and migration-related exclusion, has been widely observed but remains insufficiently explored from an integrated perspective [9]. Yet, few studies have empirically tested these interconnections within a syndemic framework, particularly in urban environments like Johannesburg, where the density and diversity of risks are heightened.

While previous studies have predominantly focused on the biomedical dimensions of TB in South Africa, most adopt a fragmented or disease-specific approach. For example, Narasimhan et al. [7] explored risk factors such as HIV infection, malnutrition, and diabetes, emphasising their biological implications for TB susceptibility and progression—with minimal focus on how these intersect with social conditions—but their research failed to assess how these interact and cluster within affected populations [6]. Similarly, Lönnroth et al. examined the impact of individual-level risk factors like smoking and alcohol use, but their work did not fully integrate the broader socioenvironmental context [10]. The research by Osman et al. (2021) [4], also provides important insights into health system weaknesses but does not explore how structural barriers combine with psychosocial stressors like stigma or food insecurity to shape TB outcomes. Moreover, few studies have applied syndemic theory to examine how multiple co-occurring epidemics and vulnerabilities amplify one another in specific urban contexts. Johannesburg, as a rapidly urbanising city, presents a unique setting where migration, unemployment, housing insecurity, HIV, and stigma often converge, yet prior research tends to analyse these factors in silos. This lack of integration limits our understanding of the real-world complexity of TB vulnerability.

However, there remains a critical gap in the literature concerning how these social determinants interact dynamically with individual behaviours and structural barriers, especially in densely populated urban areas like Johannesburg. While clinical interventions have improved diagnostic capacity and treatment protocols, they fall short in terms of addressing the broader systemic drivers of disease. Research on the social determinants of TB is growing, but there remains a critical gap in understanding how these determinants interact dynamically with individual risk behaviours and structural barriers, particularly in urban, high-density environments like Johannesburg [11].

This study addresses that gap by taking an integrated syndemic approach that includes individual behaviours, social environments, and structural inequalities. By combining quantitative evidence with the Host–Agent–Environment framework and syndemic theory, it offers a more holistic and context-sensitive analysis of TB risk among people living in Johannesburg, South Africa. The study adopts a cross-sectional quantitative design, collecting data from tuberculosis patients attending selected clinics across Johannesburg. As such, the study focuses on identifying associations between behavioural, social, and structural determinants of TB rather than establishing causality.

## 2. Materials and Methods

### 2.1. Study Design

The study employed a quantitative, cross-sectional survey design to assess the syndemic relationship between individual, social, and structural determinants of tuberculosis (TB) among adults residing in Johannesburg, South Africa. The study design was chosen for its suitability in providing a snapshot of the population at a specific time and detecting associations among multiple variables [12]. This approach was particularly valuable in capturing the interconnected nature of behavioural, social, and structural risk factors contributing to TB burden.

### 2.2. Study Setting

The research was conducted in Johannesburg. Four administrative regions were randomly selected from the clinics treating patients with tuberculosis in Johannesburg to ensure demographic diversity, and data were collected from six Community Healthcare Centres (CHCs) providing TB care, based in Soweto, Alexandra, and other parts of Johannesburg.

### 2.3. Sampling Strategy and Study Population

The target population for this study comprised adults aged 18 years and older who were currently undergoing treatment for tuberculosis at public health clinics in Johannesburg. A three-stage cluster sampling design was employed to recruit study participants. The study population consisted of adults (aged 18 years and older) living with tuberculosis (TB) in Johannesburg, South Africa. The sampling focused on individuals receiving treatment at public health clinics administered by the City of Johannesburg. Four administrative regions (out of the seven in the Johannesburg Metropolitan Municipality) were randomly selected. In Stage 1, four of Johannesburg’s seven administrative regions (approximately 57%) were randomly selected using a simple random sampling technique from the full list of regions where public TB clinics operate to ensure geographic, socioeconomic, and demographic diversity. In Stage 2, two public health clinics providing TB treatment were purposively selected from each of the four chosen regions, yielding a total of six clinics. These clinics were selected from the 81 City of Johannesburg-administered primary healthcare facilities based on their accessibility, high TB caseloads, and operational capacity during the study period. The selection aimed to include approximately 10% of clinics treating TB patients in the targeted regions to ensure variation in patient demographics and service environments. In Stage 3, systematic sampling was used to recruit TB patients attending the selected clinics over a four-week data collection period. Eligible participants were adults diagnosed with TB and receiving treatment at the time of the study. Clinic TB registers served as the sampling frame, and patients were selected at regular intervals based on daily attendance patterns until the target sample size of 239 participants was reached. The sample size (*n* = 239) was determined using the standard formula $Sample\;Size=\;\frac{{\left(Z\right)}^{2}\;x\;p(1-p)}{{\left(dl\right)}^{2}}$ for population-based surveys, where *Z* is the z-score (1.96 for 95% confidence), *p* is the estimated proportion of the attribute in the population (assumed to be 0.5 to maximise variability), and *d* is the margin of error (set at 0.05). This assumption of *p* = 0.5 is widely accepted in health research when no reliable prior estimates exist, as it ensures the maximum required sample size for conservative precision [13]. Although the initial calculation yielded a higher value (~384), the final sample size was adjusted to 239 participants in line with logistical constraints and based on expected design effect and participant availability at selected clinics, as discussed in similar studies.

### 2.4. Data Collection and Analysis

The data were collected using a structured questionnaire covering the individual, social, and structural determinants of TB. Data were collected at six selected clinics during TB clinic operating hours. Participants were recruited while they were waiting in line to be attended to. Each participant received an information and consent letter, which they were asked to complete prior to participation. Once consent was obtained, participants were taken to a private room where the data collection process was conducted in a confidential setting. The data were computed using the Statistical Package for the Social Sciences (SPSS) version 29.0.0.0 software and processed to obtain graphical representations. The study can be classified as a multivariate study, and hence, factor analysis was used. For analytical reasons, all variables chosen for the study were factorised based on age groups using the Principal Component Analysis (PCA) technique. Syndemic theory was also used to analyse the syndemic interaction between key social and structural factors and tuberculosis. The syndemic theory was used to analyse cross-sectional associations between individual factors (smoking, alcohol, substance abuse, unemployment, poverty, inequality, HIV/AIDS, and tuberculosis) and the occurrence of tuberculosis infection among people living in Johannesburg.

### 2.5. Ethical Considerations

Ethical clearance for this study was obtained from the University of Johannesburg Faculty of Health Sciences Research Ethics Committee (REC), approval number: REC-241112-035. Additional permission to conduct the research in municipal health facilities was granted by the City of Johannesburg Health District (reference number: 2023-10-04-JHBHealth). All participants were provided with written information about the study and signed an informed consent form prior to participation. Participation was voluntary, and respondents were assured of confidentiality, anonymity, and the right to withdraw at any stage without any consequences. The study adhered to the ethical guidelines set out in the Declaration of Helsinki and complied with the university’s standards for human subject research.

## 3. Results

### 3.1. Demographic and Socioeconomic Characteristics of Participants

The study included 239 tuberculosis (TB) patients from six selected clinics in Johannesburg. The gender distribution showed a higher proportion of males (60.3%) compared to females (39.7%). In terms of age, the participants ranged from 20 years and older, with the largest age group being 30–39 years (38.1%), followed by 40–49 years (29.3%) and 50–59 years (15.1%). Only a small percentage (3.3%) were aged 60 years and above. Table 1 shows the demographic characteristics of the study population.

Most participants identified as African (96.7%), with minority representation from White (1.3%), Coloured (1.7%), and Indian/Asian (0.3%) groups. Regarding marital status, the majority were married (64.9%), followed by divorced or separated (19.2%), never married (13.8%), and widowed (2.2%). In terms of employment, 50.2% (*n* = 120) were unemployed, while 47.7% (*n* = 114) were employed or self-employed, and 2.1% (*n* = 5) were students.

All participants reported some level of formal education. The majority had completed high school (70.3%, *n* = 168), while 13.4% (*n* = 32) had a college qualification, and 7.1% (*n* = 17) had attended university. A smaller portion (9.2%, *n* = 22) had only primary-level education. When asked about income in the previous month, 67.4% (*n* = 161) reported that they received an income—primarily from salaries (47.3%, *n* = 113) or contributions from family (29.3%, *n* = 70).

Among participants who reported receiving an income, the most frequently reported income bracket was ZAR 48,001 to ZAR 96,000 per year (23.4%, *n* = 56), which is equivalent to a monthly income of ZAR 4001 to ZAR 8000. However, this did not represent the majority. When examining the overall distribution, the median income group fell within the ZAR 12,001 to ZAR 24,000 range (11.3%, *n* = 27), suggesting that a significant portion of participants earned at the lower end of the income scale and indicating a lower income bracket. Table 2 shows the distribution of income of the participants.

### 3.2. Individual and Behavioural Factors for TB Acquisition

The study also explored the HIV status of participants, given the well-documented link between TB and HIV. More than half of the participants (56.1%, *n* = 134) indicated that they were HIV-positive, while 43.9% (*n* = 105) were HIV-negative. The distribution is shown in Table 3.

The study assessed alcohol and tobacco use as key individual behavioural risk factors for TB acquisition. Among the 239 participants, 45.2% (*n* = 107) reported having consumed alcohol, while 55.2% (*n* = 132) stated they had never consumed it. Of those who drank alcohol, the majority (56.1%, *n* = 60) consumed alcohol 2 to 4 times per month, followed by 22.4% (*n* = 24) who drank 2 to 3 times per week and 19.6% (*n* = 21) who drank once a month or less. Only one participant (0.93%) reported drinking four or more times a week, and one participant (0.93%) had not consumed alcohol in the past 12 months. Most alcohol users (64.8%, *n* = 70) typically consumed one or two drinks per drinking occasion, while 33.3% (*n* = 36) reported consuming three or four drinks. No participants reported drinking more than four drinks at a time. Table 4 shows the frequency of alcohol consumption in the past 12 months.

In terms of tobacco use, 33.1% (*n* = 79) of the participants indicated that they smoked, whereas 66.9% did not. Among the smokers, nearly all (97.5%) smoked daily, and only 2.5% reported smoking less than daily, as shown in Table 5.

### 3.3. Social and Environmental Factors Enabling the Transmission of Tuberculosis

The study explored household size, living conditions, and residential areas as key social and environmental contributors to TB transmission. Among the 239 participants, 43.5% (*n* = 104) reported that they lived in households with 4–5 people, while 24.7% (*n* = 59) lived with 6–7 people. Notably, 10.5% (*n* = 25) resided in overcrowded households with eight or more individuals, and only 4.6% (*n* = 11) lived alone.

In terms of housing infrastructure, 19.2% (*n* = 46) of participants lived in one-room dwellings, while 43.9% (*n* = 105) lived in homes with four rooms, and 27.6% (*n* = 66) had five or more rooms. The majority (82.8%, *n* = 198) reported living in brick houses, while 17.2% (*n* = 41) lived in tin houses. Additionally, the length of time participants had lived in their current residence varied: 29.7% (*n* = 71) had lived there for 11–15 years, and 22.2% (*n* = 53) for 16 years or more. Most participants (81.2%, *n* = 194) resided in high-density suburbs, with 13.8% (*n* = 33) living in medium-density and only 5% (*n* = 12) in low-density areas. In terms of household composition, 63.2% (*n* = 151) lived with 0–1 children, 32.2% (*n* = 77) with 2–3 children, and 4.6% (*n* = 11) with 4–5 children. When asked about TB cases in the household, 98.3% (*n* = 234) reported being the only affected person, while 1.7% (*n* = 4) indicated that another household member also had TB. Table 6 shows the distribution of household sizes among participants.

### 3.4. Structural Factors Enabling the Transmission of Tuberculosis

Structural factors such as childhood vaccination, history of TB diagnosis, and access to treatment services play a critical role in understanding TB transmission patterns in Johannesburg. In this study, all participants (*n* = 172) who had children confirmed that their children had received the BCG vaccination. Regarding disease history, the largest proportion of participants (45.6%, *n* = 109) had been diagnosed with TB 2–3 months prior to the study. Another 31.0% (*n* = 74) had been diagnosed 4–5 months ago, and 16.7% (*n* = 40) were recently diagnosed within the last 2–4 weeks. A smaller group (6.7%, *n* = 16) had been living with a TB diagnosis for six months or more. Similarly, the duration of TB medication use mirrored the diagnosis timeline. Most participants (45.6%, *n* = 109) had been on treatment for 2–3 months, 31.4% (*n* = 75) for 4–5 months, and 16.7% (*n* = 40) for 2–4 weeks. A small number (6.3%, *n* = 15) had been taking medication for six months or longer. The distribution of structural factors is shown in Table 7.

### 3.5. Stigma and Adherence to TB Medication

The study explored the role of stigma in TB medication adherence. All the participants indicated that they shared their diagnosis with someone outside their household after being diagnosed with TB. However, 24% (*n* = 58) of participants indicated that they were teased or sworn at after their diagnosis. Additionally, 17.6% (*n* = 42) mentioned feeling unclean or dirty following their diagnosis, whereas 29.3% (*n* = 70) indicated that they were gossiped about, reflecting the emotional and psychological burden associated with TB.

### 3.6. Migration Patterns and Experiences of the Study Participants

The study examined the migration backgrounds and experiences of the 239 participants. The majority 82.8% (*n* = 197) were South African-born, while 15.1% (*n* = 36) originated from other Southern African countries, and 2.5% (*n* = 6) were from East and Central African countries such as Somalia, Ethiopia, Congo, or Cameroon. Among the participants not born in South Africa, 33.4% had lived in the country for 13 years or more, 28.6% for 10–12 years, and smaller groups for 7–9 years (19%), 4–6 years (9.5%), and 1–3 years (9.5%). Regarding immigration status, 54.8% were documented migrants, 26.2% were undocumented, and 19% identified as asylum seekers. Table 8 shows the migration patterns and experiences of the study participants.

### 3.7. Testing the Syndemic Relationship Between Individual and Social Risk Factors for Tuberculosis

To explore how individual behaviours and social conditions interact to influence tuberculosis risk, the study employed Principal Component Analysis (PCA). This statistical method was used to group related variables and determine how much of the variability in the data could be explained by underlying factors. The analysis extracted four key components, which together explained 78.8% of the total variance. These components captured different combinations of individual and social risk factors.

The first component accounted for 30.6% of the total variance and was dominated by economic variables, including employment status (loading: 0.879) and gross annual income (0.930). The second component explained 19.9% of the variance and was associated with alcohol consumption (0.717), tobacco use (0.678), and housing type (0.602). The third component, responsible for 16.6% of the variance, captured substance use and housing density, with significant loadings for the number of rooms in the house (−0.574, 0.544), smoking (0.603), and alcohol use (0.540). The fourth component, which explained 11.7% of the variance, was linked to health access and status, with strong loadings for HIV status (0.709) and place of healthcare (0.721).

Additionally, the communality values showed how well each variable was represented by the extracted components. Table 9 indicates the proportion of variance in each variable that is explained by the extracted factors. A higher communality value suggests that the variable is strongly associated with the factors identified in the analysis. In this study, HIV status had a communality value of 0.578, indicating that 57.8% of its variability is explained by the extracted factors. Individual-level variables like alcohol use (0.835) and smoking (0.839) were well represented by the factors, as were social risk factors such as employment situation (0.822) and gross annual income (0.932), highlighting strong links to economic stability. Housing-related variables, including type of housing (0.856) and number of rooms in the house (0.848), also had high communalities, showing the significance of living conditions. Healthcare access showed a moderate communality (0.569), suggesting partial representation by the factors, with some influence from external elements.

## 4. Discussion

### 4.1. Individual and Behavioural Risk Factors Associated with TB Infection

The study revealed that individual and behavioural risk factors such as smoking, alcohol consumption, and HIV co-infection play a significant role in TB transmission. Of the total participants, 33.1% of participants were smokers, and 45.2% consumed alcohol, aligning with the existing literature that links these behaviours to increased TB risk [7]. Smoking damages lung function, making individuals more vulnerable to infection [14], while 97.7% of smokers in the study smoked daily, exacerbating this risk [15,16]. Additionally, 56.1% of participants were HIV-positive, reinforcing the established association between HIV and TB, as co-infection severely compromises the immune system and accelerates disease progression [11,17,18].

Alcohol consumption was also found to be a major contributor to TB vulnerability, with 56.1% of alcohol users drinking two to four times per month. Excessive alcohol use weakens the immune system and is linked to delayed diagnosis and poor treatment adherence [7,19,20]. Furthermore, poor nutrition emerged as an underlying behavioural risk factor, with 67.4% of participants depending on low-income sources like family contributions or social grants. Food insecurity and malnutrition impair immune responses, increasing TB susceptibility and worsening health outcomes, as supported by Lönnroth et al. [10] and Balinda et al. [21]. These findings highlight the compounded effects of behavioural and socioeconomic vulnerabilities on TB infection risk.

### 4.2. Social and Environmental Factors Enabling TB Transmission

The study highlighted several social determinants such as unemployment, poverty, overcrowding, and poor housing conditions that contribute to TB transmission. Half of the participants (50.2%) were unemployed, and 29.3% earned less than ZAR 4000 per month, reflecting economic instability that limits access to healthcare and nutritious food, thereby increasing TB vulnerability [5]. Household size also emerged as a significant factor, with 43.5% living in homes with 4–5 people and 10.5% in overcrowded conditions with 8 or more people, which supports existing evidence that overcrowding and prolonged close contact increase TB transmission risk [6,10]. Additionally, 19.2% of participants lived in one-room dwellings, indicating inadequate living conditions that hinder TB prevention.

Housing type further illustrated the environmental risks, with 82.8% living in brick houses and 17.2% in tin houses, with the latter often found in informal settlements lacking proper sanitation and healthcare access [5]. These findings support the previous literature linking urban poverty and inadequate housing infrastructure to high TB incidence [1]. The study also identified stigma and discrimination as critical social determinants, with 24% of participants experiencing teasing or rejection and 29.3% reporting gossip due to their TB status. This aligns with findings by Harling et al. [22], who argue that stigma delays health-seeking behaviour, reduces treatment adherence, and worsens TB outcomes by increasing isolation and emotional distress.

### 4.3. Structural Determinants of TB in Johannesburg

The study identified key structural determinants, including limited healthcare access, migration status, and systemic inequalities, as major influences regarding TB prevalence in Johannesburg. A large majority of participants (95%) relied on public health facilities, indicating restricted access to private healthcare. This aligns with Osman et al. [4], who noted that in resource-limited settings, such barriers contribute to delayed TB diagnosis and treatment, increasing the risk of ongoing transmission. The study also revealed that 54.8% of non-South African participants were documented migrants, while 26.2% were undocumented, highlighting the challenges non-citizens face in accessing healthcare. These findings reflect broader concerns raised by Narasimhan et al. [7] and WHO [1], who found that migrants often encounter legal and financial obstacles, including fear of deportation, which can delay treatment and exacerbate TB spread within these vulnerable populations.

Economic inequality was further underscored by the fact that 94.6% of participants did not have medical aid or health insurance, limiting their ability to seek prompt and adequate healthcare services. According to the WHO [16], high out-of-pocket costs for TB care serve as a significant barrier for low-income populations, frequently resulting in delayed health-seeking behaviour.

Migration status significantly intersects with other social determinants to compound TB risk. In this study, 26.2% of non-South African participants were undocumented migrants, a group particularly vulnerable due to limited access to formal employment, social services, and healthcare. These barriers often result in lower household income, overcrowded housing, and delayed healthcare-seeking behaviour [23]. The study found that among non-South African-born participants, a substantial proportion had resided in South Africa for over 10 years, yet many remained undocumented or had asylum status, suggesting long-term exclusion from formal socioeconomic participation. This prolonged marginalisation, coupled with high unemployment (50.2%) and low income (with 29.3% reporting no income), highlights the syndemic interaction between legal precarity, poverty, and TB risk [24].

The syndemic theory underscores that it is not merely the presence of these factors but their interactive and reinforcing nature that exacerbates TB vulnerability [8]. Migrants with unstable legal status may avoid clinics out of fear of deportation, further delaying diagnosis and increasing community transmission. Additionally, their income insecurity often limits their ability to afford transport to clinics, nutritious food, or adequate housing, all of which are protective factors against TB. These findings align with other research in South Africa, which shows that undocumented migrants frequently face systematic exclusion from TB prevention and treatment programmes [25].

### 4.4. Testing and Validating the Syndemic Relationship Between Individual and Social Risk Factors for TB

The study applied syndemic theory to explore how individual behaviours and social conditions interact to worsen TB outcomes. Factor analysis confirmed a syndemic relationship between behavioural risk factors such as smoking, alcohol use, and substance abuse and social determinants like poverty, unemployment, and overcrowding. Participants who smoked or consumed alcohol were more likely to be unemployed, living in overcrowded households, and experiencing food insecurity, which reflects findings by Hargreaves et al. [5] and Singer et al. [8], who documented how overlapping health and social vulnerabilities amplify TB risk and severity.

Further analysis showed that individuals engaging in high-risk behaviours also had limited access to healthcare and faced financial instability, which is consistent with Lönnroth et al. [10] and Balinda et al. [21], who highlighted that economic hardship constrains access to proper nutrition, treatment, and safe housing. These behaviours were especially common in informal settlements and under-resourced communities, reinforcing Narasimhan et al.’s [7] findings on the impact of urban structural inequality. Additionally, the study found that 56.1% of participants were HIV-positive, underscoring how HIV co-infection compounds TB vulnerability among socioeconomically disadvantaged groups [18]. The combination of these intersecting factors provides strong evidence for a syndemic relationship, where behavioural and social risks interact to increase TB incidence and worsen health outcomes.

These findings deepen our understanding of how behavioural risks and social deprivation reinforce one another to increase TB vulnerability. However, in Johannesburg, the syndemic interplay is further intensified by the city’s distinctive sociospatial and policy context. The legacy of apartheid-era urban planning has resulted in persistent spatial inequalities, with fragmented service delivery, informal settlements, and overcrowded inner-city housing, particularly in areas like Hillbrow, Alexandra, and parts of Soweto [4,22]. These environments create ideal conditions for airborne transmission, including poor ventilation, household crowding, and limited access to quality healthcare.

Furthermore, Johannesburg’s status as South Africa’s economic centre has led to high rates of both internal and cross-border migration. Yet public health and social protection systems remain poorly adapted to accommodate the unique needs of migrant populations. In this study, undocumented migrants reported restricted access to TB services due to fear of deportation, legal exclusion, and stigma—barriers well documented in previous research [4,23,24]. These dynamics illustrate how structural and legal frameworks shape the lived experience of health vulnerability, extending the syndemic interaction beyond co-morbidities to include institutional exclusion.

In this context, our findings contribute to syndemic theory by empirically demonstrating that syndemics are not merely biomedical interactions but are deeply embedded in the urban political economy. Johannesburg’s TB epidemic reflects the interlocking nature of behavioural, social, and structural determinants that are exacerbated by urban governance gaps, migrant marginalisation, and spatial poverty [8,9]. To effectively respond, TB control efforts must expand beyond biomedical approaches to include multisectoral interventions targeting housing, employment, migration policy, and urban infrastructure [5,16]. These should include mobile health units in informal settlements, expanded legal access to healthcare for non-citizens, and integrated social protection schemes for unemployed and low-income populations.

### 4.5. Strengths and Limitations of the Study

One of the key strengths of this study lies in its use of a quantitative cross-sectional design, which enabled the collection of rich, real-time data from a diverse sample of tuberculosis (TB) patients across multiple clinics in Johannesburg. The study’s incorporation of individual, social, and structural determinants, along with the application of syndemic theory and multivariate analysis techniques such as factor analysis, allowed for a comprehensive understanding of the complex interactions influencing TB transmission. Ethical standards were also followed carefully, with official approvals obtained and participant confidentiality protected throughout. However, the study also has some limitations. Due to the study’s cross-sectional nature, it restricts causal inferences, as it captured associations at a single point in time. The research was also conducted only within Johannesburg, limiting the generalisability of the findings to other areas in South Africa or beyond.

## 5. Conclusions

This study confirms that tuberculosis (TB) in Johannesburg is shaped by the co-existence and interaction of individual, social, and structural factors. High rates of smoking, alcohol use, and HIV co-infection among TB patients point to the urgent need for targeted behavioural interventions. At the same time, social challenges such as poverty, unemployment, overcrowding, and stigma contribute significantly to the transmission of TB and delays in seeking treatment. Structural issues including limited healthcare access, substandard housing, and the vulnerable status of migrants further intensify the burden of TB. These findings call for a multisectoral approach that combines health, social welfare, and economic development strategies.

The research aligns with the syndemic framework, demonstrating how TB risk factors cluster and intersect with broader social and economic conditions. The study adds to existing evidence that adverse conditions such as malnutrition, substance abuse, poverty, and poor access to healthcare interact to magnify disease outcomes [5,8,10]. It also highlights that economic hardships, like low income and unemployment, limit healthcare access and delay diagnosis, reinforcing findings from Narasimhan et al. [7] on the disproportionate impact of TB on marginalised groups.

Additionally, the study underscores the role of stigma and discrimination in discouraging early treatment and worsening health outcomes. These findings are consistent with Courtwright and Turner [26], who identified stigma as a major barrier to TB control. The study also reveals that undocumented migrants face substantial challenges in accessing TB services, which may contribute to delays in diagnosis and treatment, potentially increasing community-level vulnerability and supporting observations by WHO [16] and WHO [1]. Overall, the study highlights the need for policy reforms and inclusive healthcare strategies that address both the biomedical and social drivers of TB, particularly for vulnerable populations.

While the findings reaffirm known risk patterns, their significance lies in illustrating how these factors interact within the Johannesburg context, where spatial inequality, migration, and policy exclusions deepen health disparities. The study provides empirical support for a syndemic approach, showing that TB vulnerability emerges not just from disease co-occurrence but from the interdependence of health, housing, employment, and legal status.

For healthcare practitioners, this means TB care should not only include clinical diagnosis and treatment but also screening for alcohol and tobacco use, food insecurity, mental health distress, and HIV co-infection. Cross-training of staff in syndemic-informed care, recognizing the impact of socioeconomic stressors on adherence and outcomes is essential. Community-based outreach and the use of mobile health services in under-served and migrant-heavy areas can also improve access and early diagnosis. For policymakers, the study reinforces the importance of inclusive health policies that address the needs of undocumented migrants and residents of informal settlements. Urban health interventions should be embedded in broader strategies to improve housing, employment opportunities, and access to legal documentation. TB control programs must be intersectoral, involving not only health departments but also departments of housing, labour, social development, and immigration.

In summary, the study establishes that TB in Johannesburg is not merely a biomedical issue but a deeply entrenched public health challenge influenced by a network of interrelated risk factors. Effective TB control efforts must extend beyond medical treatment to encompass broader social, economic, and policy interventions. By understanding and addressing the syndemic relationships between TB and its risk factors, policymakers, healthcare providers, and community organisations can work together to develop more effective and sustainable TB prevention and treatment strategies. Future research should explore additional structural determinants, including urban planning and economic policies, to develop more targeted interventions tailored to the specific needs of affected populations. Future studies should also explore the syndemic dynamics of TB using longitudinal designs to assess how structural and behavioural risks evolve over time and affect treatment outcomes. There is also a need for intervention-based research that evaluates integrated care models, such as TB, HIV and substance use co-management or mobile outreach in informal settlements. Comparative studies across other South African cities could further highlight how different urban and policy environments shape syndemic vulnerability.

## Figures and Tables

**Table 1:** Demographic characteristics of the study population.

Demographics		Frequency	Percentage
Gender	Male	144	60.3
	Female	95	39.7
Age Group	20–29 years	34	14.2
	30–39 years	91	38.1
	40–49 years	70	29.3
	50–59 years	36	15.1
	60 years and above	8	3.3
Marital status	Married	155	64.9
	Never Married	33	13.8
	Divorced/Separated	46	19.2
	Widowed/Widow	5	2.2
Family Employment	Unemployed	120	50.2
	Student/Pupil/Learner	5	2.1
	Employed/Self-employed	114	47.7
Race	African	231	96.7
	White	3	1.3
	Coloured	4	1.7
	Indian/Asian	1	0.3
Highest Educational Level	Primary School	22	9.2
	High School	168	70.3
	College	32	13.4
	University	17	7.1

**Table 2:** Income distribution of study participants.

Gross Annual Income Before Tax	Frequency	Percent
No income	70	29.3
Less than ZAR 6000 per year (Less than ZAR 500 per month)	12	5.0
ZAR 6001–ZAR 12,000 per year (ZAR 501 to ZAR 1000 per month)	25	10.5
ZAR 12,001–ZAR 24,000 per year (ZAR 1001 to ZAR 2000 per month)	27	11.3
ZAR 24,001–ZAR 48,000 per year (ZAR 2001 to ZAR 4000 per month)	37	15.5
ZAR 48,001–ZAR 96,000 per year (ZAR 4001 to ZAR 8000 per month)	56	23.4
ZAR 96,001–ZAR 192,000 per year (ZAR 8001 to ZAR 16,000 per month)	10	4.2
ZAR 192,001–ZAR 384,000 per year (ZAR 16,000 to ZAR 32,000 per month)	2	0.8

**Table 3:** HIV status among TB patients in Johannesburg.

HIV Status	Number of Participants with TB	Percent
Positive	134	56.1
Negative	105	43.9

**Table 4:** Frequency of alcohol consumption in the past 12 Months.

Frequency of Having a Drink Containing Alcohol in the Past 12 Months	Frequency	Percent
Not in the past 12 months	1	0.93
Once a month or less	21	19.6
2–4 times a month	60	56.1
2–3 times a week	24	22.4
4 or more times a week	1	0.93

**Table 5:** Tobacco use.

Tobacco Use	Frequency	Percent
Yes	79	33.1
No	160	66.9
Total	239	100
Smoked daily	77	97.5
Smoking less than daily	2	2.5
Total	79	100

**Table 6:** Frequency distribution of social and environmental factors.

Social and Environmental Factors		Frequency	Percentage
How many people live in your house including yourself?	1	11	4.6
	2–3	40	16.7
	4–5	104	43.5
	6–7	59	24.7
	8 or more	25	10.5
How many rooms does the house have?	1	46	19.2
	2	14	5.9
	3	8	3.3
	4	105	43.9
	5 or more	66	27.6
How many children are you living with?	0–1	151	63.2
	2–3	77	32.2
	4–5	11	4.6

**Table 7:** Structural factors enabling the transmission of TB.

Structural Factors		Frequency	Percentage
Childhood vaccination	Children had received the BCG vaccination	172	100
History of TB diagnosis	Were recently diagnosed within the last 2–4 weeks	40	16.7
	Had been diagnosed with TB 2–3 months prior to the study.	109	45.6
	Had been diagnosed 4–5 months ago	74	31
	Had been living with a TB diagnosis for six months or more	16	6.7
Access to treatment services	Had been on treatment for 2–4 weeks	40	16.7
	Had been on treatment for 2–3 months,	109	45.6
	Had been on treatment for 4–5 months,	75	31.4
	Had been taking medication for six months or longer	15	6.3

**Table 8:** Migration patterns and experiences of the study participants.

Migration Pattern	Frequency	Percent
South African-born	197	82.8
Originated from other Southern African countries	36	15.1
Originated from East and Central African countries such as Somalia, Ethiopia, Congo, or Cameroon	6	2.5
Among Non-South Africans, those that had lived in the country for:		
13 years or more	14	33.4
10–12 years	12	28.6
7–9 years	8	19.0
4–6 years	4	9.5
1–3 years	4	9.5
Documented migrants	23	54.8
Undocumented migrants	11	26.2
Asylum seekers	8	19.0

**Table 9:** Communalities of individual and social risk factors for tuberculosis from factor analysis.

Communalities	Initial	Extraction
HIV Status	1.000	0.578
How would you describe your present employment situation?	1.000	0.822
Have you ever had a drink containing alcohol?	1.000	0.835
Do you currently smoke tobacco?	1.000	0.839
Did you receive an income from any source in the last month?	1.000	0.815
What is your gross annual income before tax?	1.000	0.932
Where do you usually obtain health care?	1.000	0.569
What type of house do you live in?	1.000	0.856
How many rooms does the house have?	1.000	0.848

## Data Availability

The datasets generated and analysed during this study are not publicly available due to confidentiality agreements. However, anonymised data, questionnaires, and analysis codes are available from the corresponding author upon reasonable request for replication or secondary analysis.

## Abbreviations

The following abbreviations are used in this manuscript:

TB	Tuberculosis
HIV	Human Immunodeficiency Virus
AIDS	Acquired Immunodeficiency Syndrome
CHC	Community Healthcare Centre
SPSS	Statistical Package for the Social Sciences
PCA	Principal Component Analysis
CSDH	Commission on Social Determinants of Health
BCG	Bacillus Calmette–Guérin (vaccine)
WHO	World Health Organization
COPD	Chronic Obstructive Pulmonary Disease
UNISA	University of South Africa
USAID	United States Agency for International Development
REC	Research Ethics Committee
NHRD	National Health Research Database
